# Encephaloid Cancer of the Left Superior Sinus; Excision of the Upper Jaw

**Published:** 1864-01

**Authors:** J. Gordon Maxwell


Encephaloid Cancer of the left superior Sinus; Excision of the upper Jaw.—*By Dr. J. Gordon Maxwell.*—Encephaloid cancer occurs in nearly all the organs of the body, but most frequently in the mamma, eye, testicle, uterus, liver, periosteum and bones in both sexes and all periods of life. Being an extremely vascular structure it is endowed with a high degree of vitality, growing with great rapidity and often attaining an uncommon bulk in a few months from its first appearance, and causing the death of the patient generally in from nine to eighteen months. As it advances ulceration sets in, and the neighboring lymphatic glands are soon involved, thus hastening it to the final termination.

The present case is that of a man 50 years of age, a patient of Dr. Stroud of Moorestown, N. J. The tumor, which occupied the left superior maxilary region, was first noticed about three months ago, and came on without any assignable cause. There was some pain of a throbbing character, but the principal trouble was the inconvenience experienced from the tumor, as it extended across the ala of the nose on one side, and also projected some distance into the mouth.

Prof. Gross, after a careful consideration of the case, determined to excise the upper jaw, as it was almost completely involved in the morbid growth.

As a sitting posture was desired during the operation, ether was administered instead of chloroform. When the patient was sufficiently influenced, Prof. Gross commenced the operation by making an incision from a little below the external angle of the eye to the commissure of the lips, the flaps were then carefully dissected up while the bleeding vessels were compressed by an assistant. The facial and coronary arteries, which had been divided, were then ligated, and as there existed no obstacle the patient having lost most of his teeth, the jaw was excised by means of the bone forceps, the whole removal not occupying half a minute. Prof. Gross then followed the remains of the morbid mass into its remotest recesses and removed the whole of it. The edges of the wound were then approximated by means of five twisted and two interrupted sutures. The cavity was stuffed with patent lint wet with persulphate of iron, which was removed and a fresh application made on the fifth day after the operation.

The wound healed by the first intention, and on the tenth day as no untoward symptom had arisen, the patient was allowed to return to his home in New Jersey.—*Medical and Surgical Reporter.*

